# Optimizing tourniquet pressures in knee arthroscopy: an international survey on current practice and a double-blind randomized trial

**DOI:** 10.1007/s00264-026-06926-9

**Published:** 2026-06-27

**Authors:** Heber Oliveira Cardoso Filho, Bruno Fajardo do Nascimento, Rodrigo Hohl, Vinicius Novais Rocha, Manoel Carlos Couto de Araujo, Rhaí A. Arriel, Gabriel Dias Mendes Andrade, Moacir Marocolo

**Affiliations:** 1 Medical Faculty, Federal University of Juiz de Fora, Juiz de Fora, Brazil; 2 Department of Orthopaedics and Traumatology, University Hospital, Federal University of Juiz de Fora, Juiz de Fora, Brazil; 3 Integrated Laboratory of Physiology and Performance (LABIFID), Department of Biophysics and Physiology, Federal University of Juiz de Fora, Juiz de Fora, Brazil; 4 Department of Morphology, Federal University of Juiz de Fora, Juiz de Fora, Brazil

**Keywords:** Tourniquet pressure, Knee arthroscopy, Postoperative pain, Analgesic consumption, Individualized occlusion pressure

## Abstract

**Background & purpose:**

Pneumatic tourniquets improve surgical visualization during knee arthroscopy, but fixed high pressures may increase postoperative pain. This study investigated whether a minimal effective individualized occlusion pressure (MEIOP), calculated as limb occlusion pressure plus a safety margin, maintains surgical field quality while reducing pain compared to a standardized occlusion pressure (SOP).

**Methods:**

A preliminary survey of 79 orthopaedic surgeons identified 350 mmHg as the routine SOP. In a subsequent double-blind randomized clinical trial, patients undergoing elective knee arthroscopy were assigned to SOP (350 mmHg) or MEIOP, defined as Doppler-derived limb occlusion pressure plus a safety margin (mean ≈ 230 mmHg). Outcomes included surgical field quality, postoperative using a visual analog scale (VAS) up to 15 days, overall 15-day pain perception, and analgesic consumption.

**Results:**

Both groups achieved 100% excellent surgical field ratings. Pain trajectories differed significantly between groups over time (group × time interaction, *p* = 0.022). The MEIOP group experienced significantly lower pain at discharge (~ 6 h; *p* = 0.013, d = 1.14) and lower overall 15-day pain perception (p = 0.043, d = 0.89). Analgesic consumption showed a consistent descriptive trend favoring MEIOP throughout follow-up.

**Conclusion:**

This proof-of-concept study suggests that individualizing tourniquet pressure using predefined MEIOP protocol preserves satisfactory surgical visualization and is associate with lower postoperative pain compared with a standard high-pressure protocol. This trial was prospectively registered at the Brazilian Clinical Trials Registry (ReBEC; RBR-4xd6cxm).

## Introduction

In orthopaedic surgery, particularly during knee arthroscopy, pneumatic tourniquets are widely used to induce vascular occlusion and provide a clear, bloodless surgical field [1]. Adequate visualization is essential for surgical precision, efficiency, and intraoperative safety [1]. However, excessive tourniquet pressure (TP) and prolonged limb ischaemia may cause neural compression, endothelial dysfunction, and hypoxic muscle injury [2], which may be aggravated after release by reperfusion-related oxidative and inflammatory responses [1], resulting in increased postoperative pain, swelling, and delayed recovery [3].

In practice, TP is often selected empirically based on institutional routines or surgeon preference rather than patient-specific assessment. Fixed high pressures remain common despite variability in blood pressure, limb circumference, and soft-tissue composition. In a survey of 151 orthopedists, 47% used 250–300 mmHg and 32% > 300 mmHg [4]; in a South African survey, mean TP was 398 mmHg [5]. Thus, pressures frequently exceed the minimum needed for arterial occlusion, increasing ischaemic stress and tissue damage risk.

A physiologically grounded alternative is individualized occlusion pressure (IOP), defined as the lowest pressure required to interrupt arterial flow at rest. However, limb manipulation during arthroscopy alters vascular dynamics, so resting IOP may not maintain occlusion throughout the procedure. The Minimum Effective Individual Occlusion Pressure (MEIOP), defined as IOP plus a safety margin, has therefore been proposed to ensure reliable occlusion while minimizing compression and ischemic burden. Trials in knee arthroplasty indicate procedures can be performed with pressures < 300 mmHg without compromising visualization [6–9], and a meta-analysis reported improved recovery and reduced postoperative pain with personalized pressures [10]. Nevertheless, evidence supporting MEIOP during knee arthroscopy remains limited.

This study included two sequential phases: (1) a survey identifying standardized occlusion pressures (SOP) and cuff specifications used in knee arthroscopy; and (2) a double-blind randomized trial comparing SOP with Doppler-derived MEIOP. We tested whether MEIOP could maintain a clear surgical field while reducing postoperative pain and analgesic consumption.

## Materials and methods

This study was conducted in two sequential phases to characterize current tourniquet practice and test an individualized tourniquet pressure (TP) protocol during elective knee arthroscopy. First, an online questionnaire (Google Forms®) was distributed to orthopaedic surgeons to document routine occlusion pressures and tourniquet specifications. Data were analyzed descriptively, and the most frequent standardized occlusion pressure (SOP) and cuff characteristics were used as the reference condition for Phase 2.

Phase 2 was a prospective, double-blind, randomized controlled trial comparing SOP with an individualized occlusion strategy. Patients were blinded because tourniquet inflation occurred under anaesthesia, and surgeons were blinded because TP was established and monitored by a team member not involved in the procedure. Participants were allocated (computer-generated random sequence, independent investigator) to: (1) SOP group, using the fixed pressure identified in Phase 1; or (2) individualized group, with pressure determined preoperatively from Doppler-derived limb occlusion pressure (LOP) plus a safety margin. The primary aim was to determine whether individualized pressure could maintain surgical field quality while reducing postoperative pain and analgesic use.

**Fig. 1:**
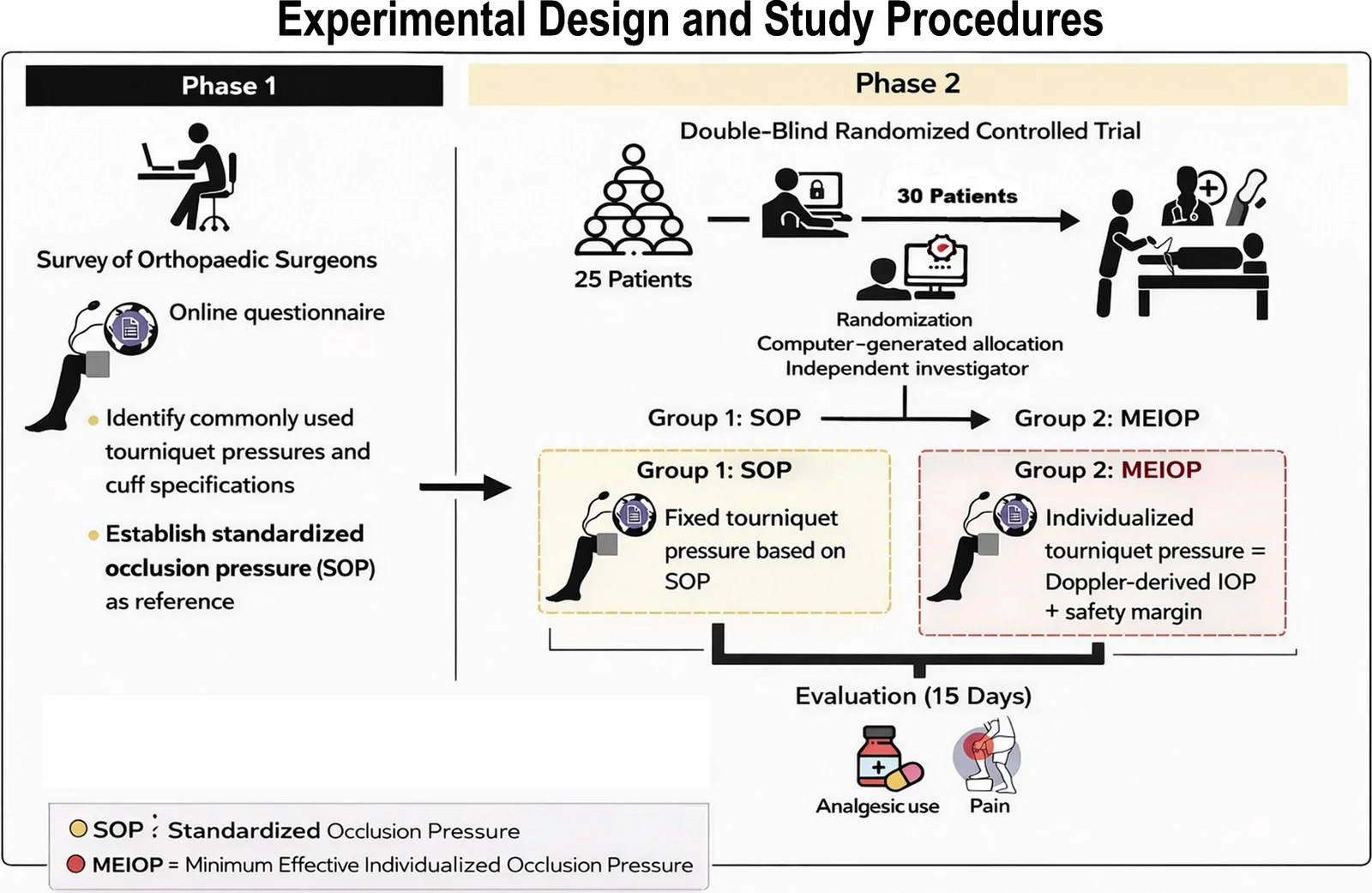
Experimental design of the study. Schematic representation of the two-phase protocol. *Phase 1* consisted of a cross-sectional survey to define the Standard Occlusion Pressure (SOP) based on current clinical practice. *Phase 2* comprised the randomized controlled trial where participants were allocated to either the SOP group or the Minimal Effective Individualized Occlusion Pressure (MEIOP) group, calculated via Doppler-derived IOP. The diagram illustrates the study timeline from recruitment and randomization to intraoperative intervention and postoperative assessment

Patients, anesthesiologists, and postoperative assessors were blinded to allocation. All procedures were performed at a university-affiliated orthopedic surgery center in accordance with the Declaration of Helsinki. The study was approved by the Local Ethics Committee of the University (approval No. 7.589.585), written informed consent was obtained from all individual participants included in the study. Additionally, the authors affirm that human research participants provided informed consent for publication of the anonymized arthroscopic images included in this manuscript. This trial was prospectively registered at the Brazilian Clinical Trials Registry (ReBEC; RBR-4xd6cxm).The study timeline is presented in Fig. 1

### Phase 1: survey of clinical tourniquet practices in knee arthroscopy

A descriptive survey was developed for this study to assess tourniquet pressures and cuff characteristics in knee arthroscopy. Content validity was reviewed by two senior orthopaedic surgeons experienced in arthroscopy and tourniquet management. The questionnaire was pilot-tested in six surgeons, and minor adjustments were made.

The final questionnaire was distributed electronically to orthopaedic surgeons through mailing lists, institutional networks, and orthopaedic societies. Surgeons from Brazil and other countries (Colombia, Germany, England, and Poland) were invited to broaden practice representation. Participation was voluntary and anonymous. The survey collected demographic/professional characteristics and detailed information regarding cuff dimensions, inflation pressures, methods for selecting pressure, and subjective surgical field quality. Results were analyzed descriptively and used to define SOP for the trial.

### Participants

Thirty adults (20 males, 10 females), aged 18–45 years, scheduled for elective knee arthroscopy were included. Baseline characteristics are shown in Table 1.

**Table 1:** Demographic and surgical characteristics of participants

Variable	SOP ~ 350 mmHg (*n* = 14)	IOP (*n* = 16)
Age (years)	31.86 ± 8.7	26.1 ± 6.0
Sex (M/F)	9/5	11/5
Individual Occlusion pressure (mmHg)	350.0 ± 0.0	172.8 ± 16.1*
Type of Surgical procedure	ACLR(9), meniscectomy/meniscal repair(2), ACLR + meniscal procedure(1), Revision ACLR(2)	ACLR(11), meniscectomy/meniscal repair(3), ACLR + meniscal procedure(2)

Values are mean ± SD or absolute frequency. *ACLR* anterior cruciate ligament reconstruction. *indicates statistical significance when compared with the SOP (*p* < *0.05)*

Inclusion criteria were: (a) elective arthroscopic procedures (meniscal repair, partial meniscectomy, ACL or PCL reconstruction); (b) no cardiovascular, metabolic, or neurological disease; (c) eligibility for spinal anaesthesia; and (d) written informed consent. Non-inclusion criteria were: (a) peripheral vascular disease or uncontrolled hypertension; (b) diabetes mellitus, coagulopathies, or autoimmune disorders; (c) surgery on the same limb in the previous 12 months; (d) infection or dermatologic lesion at cuff site; (e) chronic analgesic or vasoactive drug use; (f) pregnancy; and (g) neuropathy or tourniquet intolerance.

### Tourniquet pressure determination: from IOP to MEIOP

A pneumatic tourniquet cuff (96 cm × 13 cm; Riester Komprimeter, Germany) was applied to the proximal thigh with cotton padding. All patients underwent surgery supine under spinal anaesthesia. In the SOP group, TP was fixed at 350 mmHg.

Before trial initiation, the individualized pressure protocol was predefined as the Minimum Effective Individualized Occlusion Pressure (MEIOP). In the individualized pressure group, limb occlusion pressure (LOP) was determined preoperatively with the participant resting using a handheld Doppler (Medpej DF7001VN) over the posterior tibial artery, with progressive cuff inflation until the arterial signal disappeared. This measurement was reconfirmed immediately before surgery after patient positioning.

For surgery, the applied pressure was not LOP alone, but prespecified MEIOP, calculated by adding a safety margin to the LOP according to published recommendations [11], given that limb position affects tourniquet pressure distribution [12]. The safety increments were: + 40 mmHg if LOP < 130 mmHg; + 60 mmHg if LOP 131–190 mmHg; and + 80 mmHg if LOP > 190 mmHg10. No participant allocated to the individualized group received a LOP-only intervention, and no changes to this pressure protocol were made after randomization.

Tourniquets were inflated immediately before incision and deflated after closure or at a maximum ischemia time of 90 min. Deflation was gradual (~ 20 mmHg/s) to minimize reperfusion-related haemodynamic changes.

### Standardized surgical procedures

All arthroscopies (meniscal repair, partial meniscectomy, or ACL reconstruction) were performed under standardized anaesthetic and surgical conditions. Spinal anaesthesia consisted of 15 mg of 0.5% bupivacaine. No intra-articular vasoconstrictive/haemostatic agents were used.

Monitoring included continuous ECG, pulse oximetry, and noninvasive blood pressure every 5 min. Recorded intraoperative variables were: (a) ischaemia time; (b) operative time; and (c) complications (excessive bleeding, reinflation need, discomfort). To reduce inter-surgeon variability, all surgeries were performed by the same orthopaedic surgeon/team. An adductor canal block was performed at the end of surgery in both groups.

### Subjective and functional outcome measurements

Surgical field quality was rated 5 min after inflation by two independent surgeons (orthopaedic residents) using a validated simplified 3-point Likert scale focused on the adequacy of visualization and haemostasis for completing the arthroscopic procedure: 1 = Excellent; 2 = Acceptable; 3 = Poor. “Excellent” or “Acceptable” was considered satisfactory. This assessment was intended to determine whether the individualized pressure strategy compromised the technical feasibility of knee arthroscopy, rather than to provide a highly granular measure of subtle differences in visualization quality.

All operated patients were included in surgical field analysis. For postoperative pain and analgesic outcomes, only patients undergoing ACL reconstruction were analyzed to avoid confounding by procedure extent. Patients with postoperative surgical site infection were excluded.

Pain intensity was assessed using a 10-cm visual analog scale (VAS) [13, 14] at discharge (~ 6 h), and at days 1 (24 h), 3, 5, 7, and 15. Patients also reported overall pain perception during follow-up.

All participants received standardized rescue analgesia: non-opioid (metamizole sodium 1 g or acetaminophen 1 g every six h as needed) and opioid (tramadol 50 mg or codeine 60 mg every six h as needed). Tramadol and codeine were converted to morphine-equivalent doses (MEDs) using an equianalgesic calculator. Patients were instructed to use a stepwise regimen (non-opioid first; opioid for severe pain). Analgesic intake was recorded as number of doses and total dosage, including average opioid and non-opioid consumption during follow-up.

Complications were monitored by cuff-site inspection after removal and a day-15 assessment for skin injury, neurological complications, and infection.

### Statistical analysis

Data are presented as mean ± SD or median (interquartile range), based on distribution (Shapiro–Wilk). Group comparisons (SOP vs. IOP) for demographic/surgical characteristics used independent-samples t test (continuous data) and chi-square or Fisher’s exact test (categorical data).

Pain at discharge and across follow-up (days 1, 3, 5, 7, and 15) was analyzed using mixed-design ANOVA with within-subject factor Time and between-subject factor Group. Sphericity was tested with Mauchly’s test and corrected using Greenhouse–Geisser when violated. Bonferroni-adjusted post hoc tests were applied as appropriate. Homogeneity of variances was assessed using Levene’s test, followed by Mann whitney t test for overall pain comparisons. Effect size was calculated using Cohen’s d and interpreted as small (0.20– < 0.50), moderate (0.50– < 0.80), or large (≥ 0.80), whereas Cohen’s *f* values were interpreted as small (0.10– < 0.25), moderate (0.25– < 0.40), or large (≥ 0.40) [15]. Significance was set at p < 0.05. Analyses were performed in SPSS 26.0 (IBM Corp., Armonk, NY, USA).

## Results

### Survey of clinical tourniquet practices in knee arthroscopy

Seventy-nine orthopaedic surgeons participated in the survey, including respondents from Poland (*n* = 7), Germany (*n* = 4), England (*n* = 3), Colombia (*n* = 4), and Brazil (*n* = 61). Regional analyses were performed only for Brazil. Brazilian responses were distributed across the Southeast (*n *= 24), South (*n* = 15), Northeast (*n* = 11), Center-West (*n* = 7), and North (*n* = 4).

In Brazil, three surgeons reported using occlusion pressures > 350 mmHg (South, Northeast, and North: *n* = 1 each). The most frequent practice was a fixed pressure of 350 mmHg (*n* = 28), particularly in the Southeast (*n* = 11) and South (*n* = 8). Pressures of 300–349 mmHg were reported by 17 surgeons, while 13 reported pressures < 300 mmHg, most commonly in the Southeast (*n* = 6), Northeast (*n* = 3), and Center-West (*n* = 3).

Among non-Brazilian respondents, all Polish surgeons (*n* = 7) reported using a 15-cm cuff with a fixed pressure of 350 mmHg. German surgeons (*n* = 4) reported pressures between 300 and 350 mmHg, also with a 15-cm cuff. Overall, 15-cm cuffs were most frequently used (*n* = 49), followed by 18-cm (*n* = 14) and 12-cm cuffs (*n* = 9). These findings indicate a prevailing use of high standardized pressures (~ 350 mmHg) and medium-width cuffs (15 cm) as shown in Table 2.

**Table 2:** Tourniquet inflation pressures and cuff widths during elective knee arthroscopy by region/country

		Tourniquet Occlusion Pressure				Tourniquet width		
Region/Country	N*	< 300 mmHg	300–349 mmHg	350 mmHg	> 350 mmHg	12 cm	15 cm	18 cm
Brazil	61	13	17	28	3	9	38	14
Poland	7	-	-	7	-	-	7	-
Germany	4	-	4	-	-	-	4	-
England	3	-	-	3	-	-	3	-
Colombia	4	-	1	3	-	-	4	-
Total	**79 (100%)**	**13(16.5%)**	**21(26.5%)**	**35(44.3%)**	**3(3.7%)**	**9(11.4%)**	**56(70.9%)**	**14(17.7%)**

“-” indicates values not reported by respondents. Data are expressed as absolute frequencies

#### Surgical field and tourniquet pressure

In the individualized group, tourniquet pressure was applied according to the prespecified MEIOP protocol, defined as Doppler-derived limb occlusion pressure (LOP) plus a predefined safety margin following the AORN Recommended Practices for the Use of the Pneumatic Tourniquet [11]. No participant in the individualized group received LOP alone. The mean applied tourniquet pressure was approximately 230 mmHg in the MEIOP group compared with 350 mmHg in the SOP group. Despite this lower applied pressure, all procedures in both groups were rated as having excellent surgical field quality, indicating that MEIOP maintained adequate intraoperative visualization. 

**Fig. 2:**
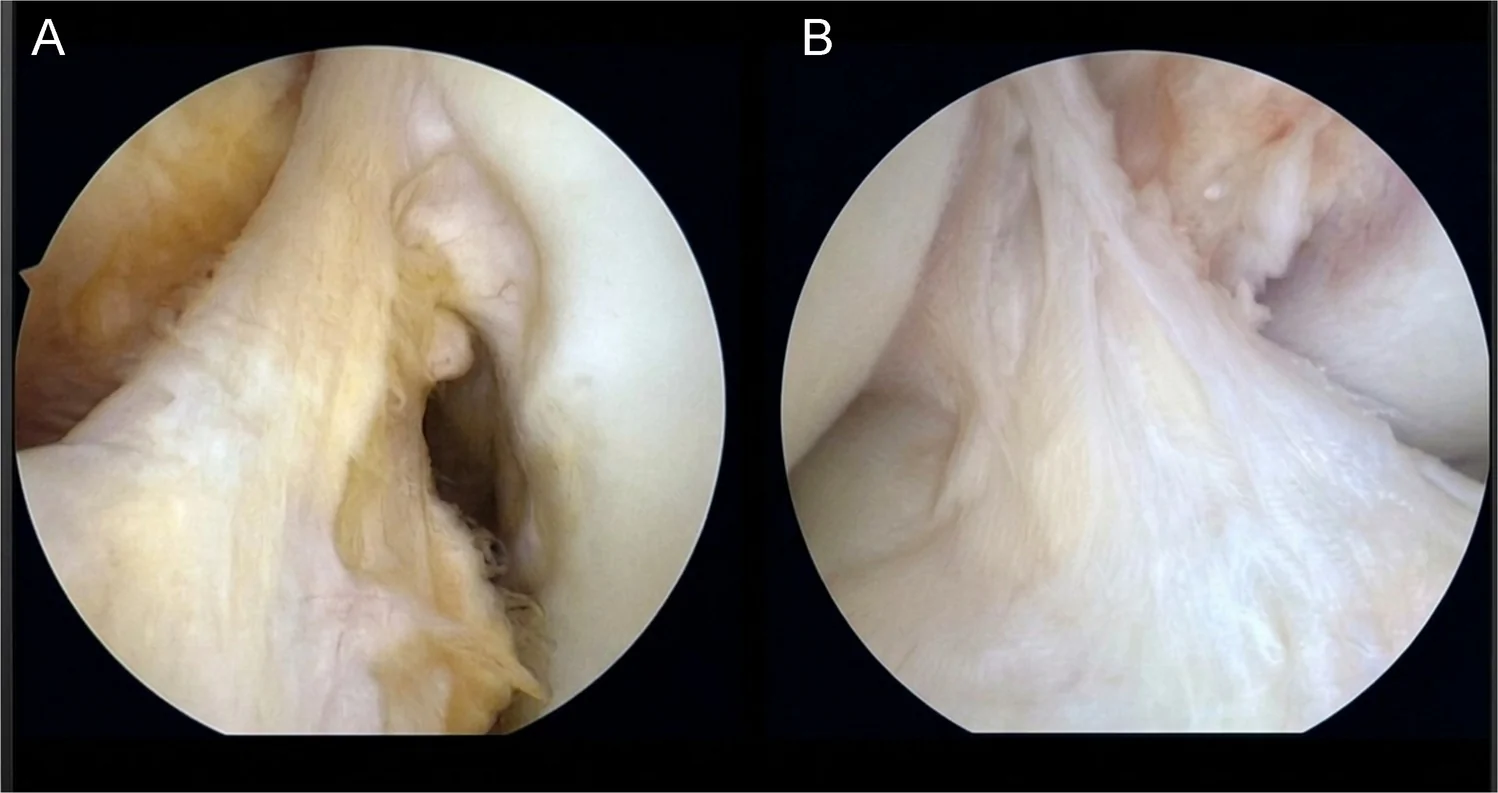
Surgical fields during knee arthroscopy using A, SOP (350 mm Hg) and B, MEIOP (~ 230 mm Hg). Images are representative and were randomly selected from the complete set of surgical field photographs obtained from all participants. Note that both conditions provided a clean surgical field

Surgical field quality was rated as excellent in 100% of cases in both SOP (350 mmHg) and MEIOP (~ 230 mmHg) groups (Table 3). Because surgical field quality was rated as excellent in all procedures, this outcome showed no variability and did not allow discrimination of subtle differences in visualization quality between pressure strategies. Representative arthroscopic images (Fig. 2) support these findings, demonstrating a clean and bloodless surgical field with both strategies.

**Table 3:** Surgical field quality ratings

Variable	SOP (*n *= 14)	MEIOP (*n* = 16)
Applied cuff pressure (mm Hg)	350 (fixed)	233.4 ± 18.5
Surgical Field Quality		
Excellent (1)	14 (100%))	16 (100%)
Acceptable (2)	0 (0%)	0 (0%)
Poor (3)	0 (0%)	0 (0%)

*SOP* standard tourniquet occlusion pressure of 350 mmHg; minimal effective individual occlusion pressure of mm Hg. *MEIOP* minimal effective individualized occlusion pressure. Field quality was evaluated using a 3-point Likert scale by the lead surgeon immediately after surgery

Pain decreased over time, with a significant main effect of Time in the two-way ANOVA (p < 0.001; *f* = 0.60). A significant Group × Time interaction was observed (p < 0.001; *f* = 0.52), indicating different pain trajectories between SOP and MEIOP.

**Fig. 3:**
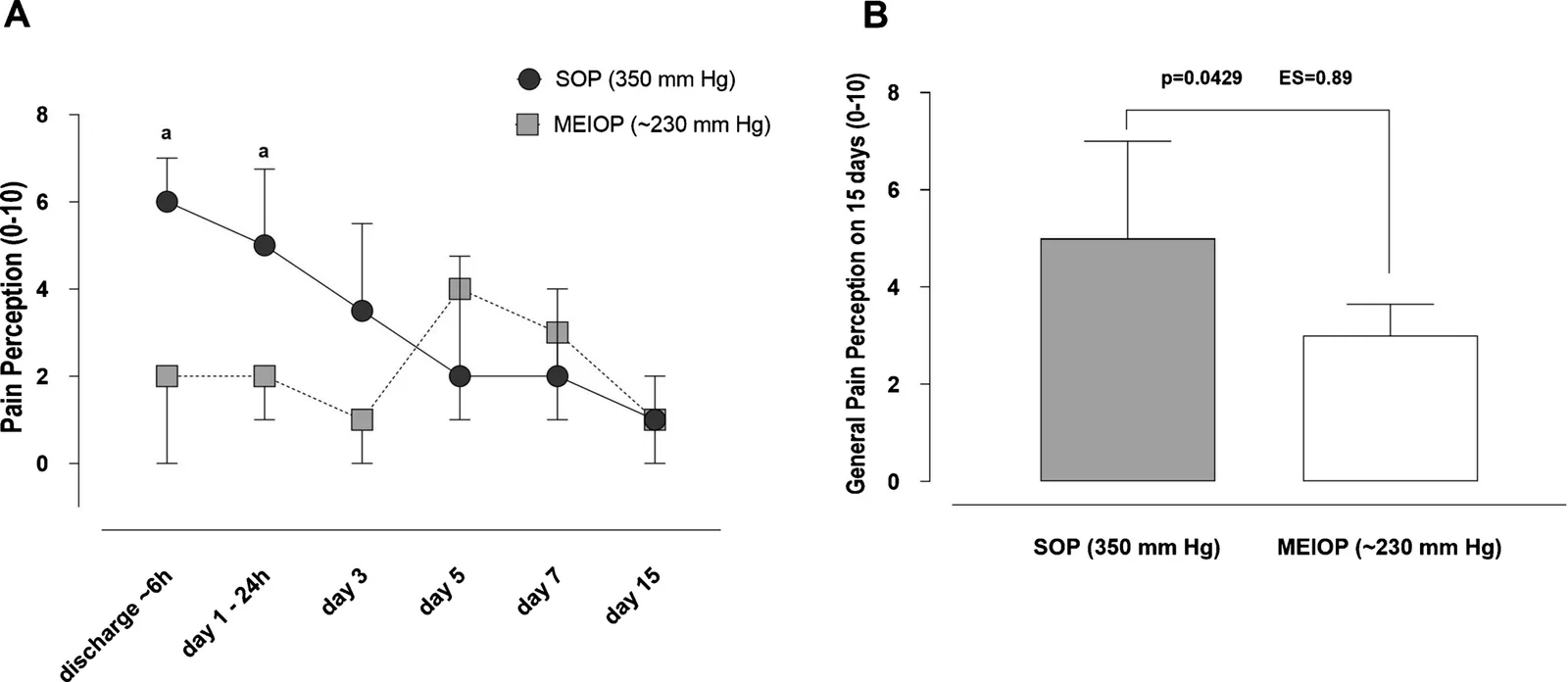
Postoperative pain intensity in the SOP (350 mmHg) and MEIOP (~ 230 mmHg) groups over the 15-day follow-up. (**A**) Pain perception across postoperative time points. (**B**) Overall pain perception during the 15-day postoperative period. ^a^p < 0.01 vs. MEIOP at the same time point

In between-group comparisons, MEIOP showed significantly lower pain at hospital discharge (~ 6 h) and day 1 compared with SOP (discharge: *p *= 0.001; d = 1.32; day 1: *p* = 0.028; d = 0.85). Pain remained numerically lower in MEIOP at day 3, with moderate effect size but without statistical significance (*p* = 0.084; d = 0.65). Overall pain over the 15-day period was significantly lower in MEIOP than SOP (Mann–Whitney U, *p* = 0.0429; d = 0.89) (Fig. 3).

**Fig. 4:**
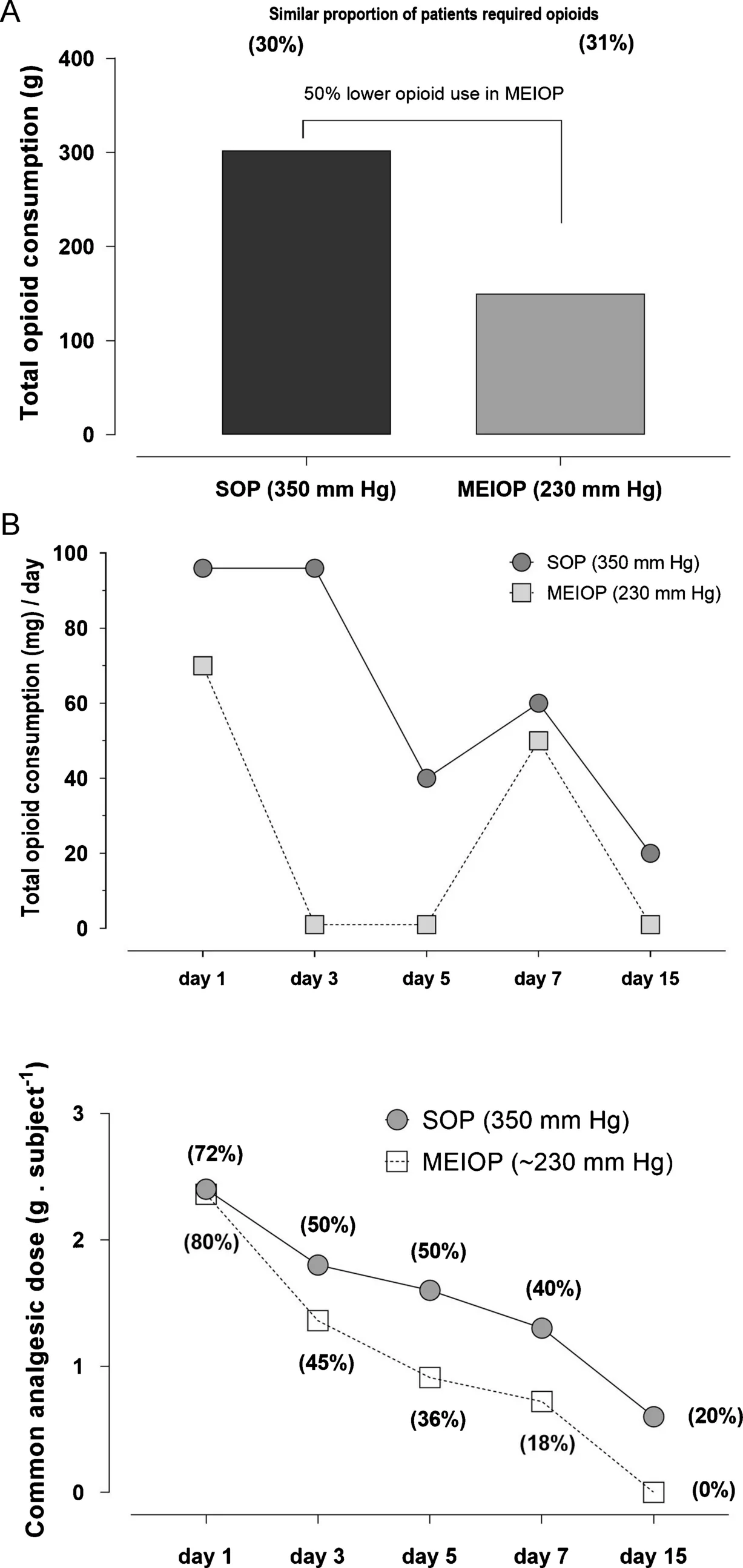
Analgesic consumption during the 15-day postoperative period. (**A**) Use of common (non-opioid) analgesics over time. (**B**) Total opioid analgesic consumption. Percentages shown in both panels represent the proportion of patients who used analgesic medication. SOP = 350 mmHg; MEIOP ~ 230 mmHg

Analgesic consumption decreased over 15 days in both groups. Visual inspection of Fig. 4 indicates consistently lower non-opioid and opioid consumption in the MEIOP group, with fewer patients requiring medication across time points compared with SOP.

## Discussion

This study provides clinically relevant proof-of-concept evidence supporting individualized tourniquet pressure strategies during elective knee arthroscopy. The survey confirmed that practice remains largely empirical and dominated by fixed high pressures, with 350 mmHg most frequently reported across regions and countries. This highlights the gap between physiological occlusion principles and routine practice, where pressure selection is standardized despite variability in blood pressure, limb circumference, and soft-tissue composition [4, 5]. The randomized phase showed that a predefined MEIOP protocol, calculated as Doppler-derived limb occlusion pressure plus a safety margin, maintained excellent surgical conditions despite substantially lower cuff pressures while improving early postoperative outcomes.

It is important to emphasize that the individualized strategy evaluated in this randomized trial was the predefined MEIOP protocol, not isolated LOP. The LOP-only approach was considered only during pre-randomization protocol development and was not applied to any randomized participant. Thus, the present findings reflect the clinical performance of an individualized LOP-based pressure-sparing strategy with an added safety margin, rather than the use of LOP alone. This distinction is clinically relevant because isolated LOP may not account for intraoperative factors that influence surgical field visualization, including limb positioning, tissue characteristics, cuff width, and changes in pressure distribution during the procedure.

Using this approach, MEIOP provided surgical visualization comparable to conventional high-pressure tourniquet inflation. Surgical field quality was excellent in 100% of cases in both protocols (Table 3), despite substantially lower cuff pressures in the individualized group. Representative arthroscopic images (Fig. 2) support preserved hemostasis and visualization with reduced pressures. This is clinically relevant because concerns about compromised visualization remain a major barrier to adopting lower pressures [16–18]. Taken together, these findings suggest that patient-tailored pressure protocols may be feasible without reliance on unnecessarily high cuff pressures. However, the surgical field quality assessment should be interpreted with caution. Because all procedures in both groups were rated as excellent, this outcome exhibited a clear ceiling effect, limiting its ability to discriminate subtle differences in visualization quality between pressure strategies. Therefore, the surgical field findings primarily indicate that MEIOP did not compromise the technical feasibility of knee arthroscopy, rather than demonstrating superiority or equivalence using a highly sensitive visualization metric. Future studies should incorporate more objective intraoperative measures, such as quantitative bleeding assessment, irrigation fluid requirements, operative time related to visualization difficulties, or digital image-based analyses of surgical field clarity.

A key methodological point is that resting individualized occlusion pressure may be insufficient in knee arthroscopy. Dynamic limb positioning alters soft-tissue tension and vascular dynamics, increasing the occlusion threshold. Early procedures showed inadequate visualization when IOP alone was applied, indicating incomplete arterial occlusion under operative conditions. After adding a safety margin according to perioperative recommendations, excellent field conditions were consistently achieved. Therefore, individualized tourniquet strategies should include a practical buffer to ensure stable occlusion during intraoperative manipulation [12].

The individualized strategy also improved early postoperative pain. Although pain decreased in both groups, the interaction indicates different trajectories. Pain was significantly lower at discharge in the individualized group, with a large effect size, and remained numerically lower in the first postoperative days. This supports that reducing pressure attenuates early pain by limiting mechanical compression and ischemic stress. Higher pressures increase tissue compression and may contribute to nerve irritation, microvascular dysfunction, and local hypoxia, while reperfusion activates inflammatory and oxidative cascades that amplify discomfort [16–19]. Applying the minimal effective pressure may reduce these stressors and improve acute recovery.

Analgesic consumption showed a consistent descriptive pattern that was directionally favourable to the individualized pressure group for both non-opioid and opioid medication. However, these findings should be interpreted cautiously because analgesic intake was self-reported, patient-driven, and analyzed descriptively. Therefore, the analgesic results should be considered exploratory and hypothesis-generating rather than definitive evidence that MEIOP reduces medication requirements.

Although reducing opioid exposure is clinically relevant for patient safety and postoperative care [20–22], the present study was not designed or powered to assess opioid-related outcomes, medication costs, or healthcare resource utilization. Similarly, while reduced analgesic requirements may have potential implications for healthcare delivery in high-volume surgical settings [23–26], larger randomized trials with prespecified analgesic outcomes, standardized medication protocols, and objective medication tracking are needed to determine whether individualized tourniquet pressure can meaningfully reduce postoperative analgesic consumption or improve resource use in knee arthroscopy.

In summary, a patient-tailored reduction in tourniquet pressure, combined with an appropriate safety margin, preserves surgical field quality while improving early postoperative pain and potentially reducing analgesic requirements. These results support shifting from empirically high fixed pressures toward individualized protocols in knee arthroscopy.

Limitations include the small sample size and the short follow-up (15 days), which precluded evaluation of longer-term functional outcomes. Although the number of participants was limited, a sensitivity analysis performed using G*Power indicated that the available sample size (n = 30) was sufficient to detect group × time interaction effects of f = 0.19 or greater with 80% statistical power at an α level of 0.05, suggesting reasonable sensitivity to detect effects of small-to-moderate magnitude. While very small effects may have remained undetected, the observed group × time interaction for pain corresponded to a large effect size (f = 0.52), substantially exceeding the minimum detectable effect estimated by the sensitivity analysis. Therefore, the study had adequate sensitive to detect the observed differences in pain trajectories over time. In addition, analgesic intake was self-reported and analyzed descriptively, and complete surgeon blinding cannot be fully ensured in tourniquet trials. Surgical field quality was also assessed using a subjective ordinal scale and showed a ceiling effect, as all procedures in both groups were rated as excellent, limiting the ability to detect subtle differences in visualization quality between pressure strategies. Because isolated LOP was not tested after randomization, the present trial does not allow direct comparison between LOP-only and MEIOP strategies. Accordingly, the findings should be interpreted as evidence for a predefined LOP-based pressure-sparing protocol with an added safety margin, rather than for the use of isolated LOP during knee arthroscopy. The present findings should be interpreted within the context of a proof-of-concept randomized trial and confirmed in larger multicenter randomized studies.

## Conclusion

This proof-of-concept randomized trial suggest that Doppler-guided individualized tourniquet pressure, applied as predefined MEIOP protocol, is a clinically feasible pressure-sparing alternative to conventional fixed high-pressure inflation during elective knee arthroscopy. MEIOP preserved excellent surgical field quality comparable to SOP (350 mmHg) while being associated with lower early postoperative pain and reduced analgesic consumption. These findings support further evaluation of lower, patient-tailored tourniquet pressures as a strategy to optimize perioperative outcomes in knee arthroscopy.

## Data Availability

The datasets generated during and/or analyzed during the current study are available from the corresponding author on reasonable request.
