# Correction to: Optimizing tourniquet pressures in knee arthroscopy: an international survey on current practice and a double‑blind randomized trial

**DOI:** 10.1007/s00264-026-06952-7

**Published:** 2026-07-31

**Authors:** Heber Oliveira Cardoso Filho, Bruno Fajardo do Nascimento, Rodrigo Hohl, Vinicius Novais Rocha, Manoel Carlos Couto de Araujo, Rhaí A. Arriel, Gabriel Dias Mendes Andrade, Moacir Marocolo

**Affiliations:** 1 Medical Faculty, Federal University of Juiz de Fora, Juiz de Fora, Brazil; 2 Department of Orthopaedics and Traumatology, University Hospital, Federal University of Juiz de Fora, Juiz de Fora, Brazil; 3 Integrated Laboratory of Physiology and Performance (LABIFID), Department of Biophysics and Physiology, Federal University of Juiz de Fora, Juiz de Fora, Brazil; 4 Department of Morphology, Federal University of Juiz de Fora, Juiz de Fora, Brazil

**Correction to: International Orthopaedics**

https://doi.org/10.1007/s00264-026-06926-9

The correct family name for the second author is Fajardo do Nascimento, rather than Fajardo. The author's full name should be displayed as Bruno Fajardo do Nascimento.

The original article has been corrected.

